# Association of Monoclonal Gammopathy of Undetermined Significance with Behcet’s Disease: A Review of Shared Common Disease Pathogenetic Mechanisms

**DOI:** 10.31138/mjr.29.2.80

**Published:** 2018-06-29

**Authors:** Ian Chikanza, Onyinye Akpenyi

**Affiliations:** Department of Rheumatology, St Barts & The Royal London Hospital, London, United Kingdom

**Keywords:** Behcet’s disease, monoclonal gammopathy, association

## Abstract

An association between a number of chronic inflammatory rheumatic diseases and Monoclonal Gammopathy of Undetermined Significance (MGUS) has been reported. To date no cases of Behcet’s disease (BD) and MGUS have been documented. BD sits at the interphase of auto-inflammatory and chronic auto-immune disease spectrums. Alterations in the cellular and cytokine microenvironments can promote a pro-inflammatory state in which persistent antigenic stimulation and cellular proliferation can progressively induce cytogenetic abnormalities which could perturbate plasma cell functions such as seen in MGUS. Herein, we present a rare case of a woman presenting with BD who subsequently developed MGUS. Pathogenetic mechanisms that could potentially contribute to development of both conditions, are reviewed and demonstrate that this disease association is not coincidental but is an evolutionary association driven by shared common disease pathogenetic mechanisms.

## INTRODUCTION

Inflammatory rheumatic diseases (RD) and neoplastic disorders can be associated in a number of ways. However, although the association of RD with a pre-malignant state such as monoclonal gammopathy of unknown significance (MGUS) has been recognised in a number of chronic inflammatory autoimmune conditions, no such link has been described in patients with Behcet’s disease.

MGUS is a CD19−, CD45− and CD56+ plasma cell disorder associated with an asymptomatic monoclonal paraproteinaemia and has a 1% average annual progression rate to multiple myeloma (MM).^1–3^ MGUS, like smoldering multiple myeloma (SMM) and MM, is a neoplastic disease that retains many of the phenotypic properties of healthy CD138+, CD19+, CD45+, CD56− plasma cells. However, MGUS plasma cells are immunophenotypically indistinguishable from those in SSM and MM, maintain low proliferation rates and can evolve into MM or Waldenström’s macroglobulinemia or AL amyloidosis or a lymphoma. There is a genomic instability associated with IgH translocations or hyperdiploidy and chromosome 13 deletion in MGUS. Its cause is unknown.

MGUS has been associated with a number of RDs: Sjogren’s syndrome (SS), Systemic Lupus Erythematous (SLE), Rheumatoid arthritis (RA) and spondyloarthropathies.^2,3^ It has been suggested that this association is not coincidental and could arise from persistent antigenic stimulation and cytogenetic abnormalities due to chronic, persistent pro-inflammatory microenvironment milieu-induced deleterious epigenetic modifications.^4^ There are no reports in the literature of an association between MGUS and BD. Given the relatively high prevalence of MGUS in the general population, the reported associations may be coincidental.^5^ However, a population-based study reported that inflammatory conditions and autoimmune diseases including rheumatic diseases, but not Behcet’s disease (BD), are significantly associated with an increased risk of developing MGUS.^6^

Behcet’s disease (BD) is a multi-systemic chronic autoinflammatory disorder exhibiting varied clinical characteristics which include the classic triad of recurrent orogenital aphthosis, uveitis, and cutaneous lesions. Nervous system, visual loss, gastrointestinal, vascular and musculoskeletal involvement may occur.^7^ From a pathophysiologic perspective, it sits at the interphase between chronic autoinflammatory and chronic autoimmune inflammatory disease, but the exact pathophysiologic mechanisms have not been fully elucidated. In BD, a pro-inflammatory state is maintained by the adaptive immune system in response to environmental and auto-antigenic factors which trigger an overexpression of pro-inflammatory cytokines and vasculitis. BD lacks the classical chronic autoimmune inflammatory disease features, such as autoantibody production, but interestingly, immunosuppressants have proven effective treatments of the condition. A unifying hypothesis has been proposed suggesting that innate and adaptive immune pathways are aberrantly integrated in BD through mechanisms such as neutrophil activation, T-cell derived chemokines and HLA-B51-associated immune reactions. Although an infectious agent probably triggers the innate-derived inflammation, bacterial persistence or autoantigen-activated antigen-presenting cells may sustain the adaptive responses.^8^

The aim of this review is to examine the association of MGUS with BD, in light of the rare case of a patient with BD who developed MGUS reported here, and to discuss the potential pathogenic mechanisms. This BD-MGUS association can provide a basis for molecular exploration of the aetiopathogenetic mechanisms of these two ill understood conditions and may offer some insights into the development of targeted therapies.

## CASE-BASED REVIEW

A 43-year-old unemployed woman, living with her husband and three children (one with Down’s syndrome) presented with arthralgia of the knee and hip joints as well as back pain. She repeated episodes of oral ulcerations, iritis, erythema nodosum and a rapidly progressive facial rash from 1993 when she was 23 years old. She also had constipation and bloating but denied shortness of breath or chest pain. She had no clinical stigmata of SLE. She also has a history of recto-colonic prolapse which was treated with colectomy. Her pathergy skin test was positive. She was diagnosed with BD which was managed with Colchicine; subsequently Anakinra, then Infliximab which induced SLE that resolved on cessation of infliximab therapy. She also tried Methotrexate and a Beclomethasone inhaler for flares of oral ulcers. The course of her illness has been one of remission and relapses.

In 2015, she was diagnosed with asymptomatic MGUS and tests performed did not indicate progression to myeloma. Serum electrophoresis showed raised IgA = 9.11g/L associated with an IgA paraprotein band, with normal IgG and IgM levels of 6.9 and 1.46g/l respectively. IgG4 levels were normal. She had normal FBC, adjusted calcium, creatinine, albumin, ESR = 8mm/Hr and CRP = <5 mg/l. Her RF, anti-CCP, ENA, ANA and anti-sDNA antibody tests were negative. HIV, Hep C and Hep B tests were also negative. Her Vitamin D_3_ levels and thyroid, cortisol and prolactin profiles were normal. She is HLA-B57.01 positive.

## DISCUSSION

We review and report here for the first time the association of BD with MGUS development. There has been a report of a patient receiving hydroxyurea who developed chronic myeloid leukaemia (CML) and three other CML cases who also developed BD whilst on IFN-α therapy.^9^ The link between RDs and MGUS has been investigated with population-based studies reporting a significant association.^5,6^ No associations of MGUS and BD have been reported in the medical literature. BD and MGUS are both complex diseases with poorly understood pathogenetic and pathophysiological mechanisms. The mechanisms involved in both conditions probably arise from shared immunological aberrations and overlap in pathogenetic pathways. One hypothesis is the shared role of chronic antigen stimulation in the aetiopathogenesis of both diseases. Autoinflammatory perturbations in BD could act as a trigger for MGUS development via epigenetic modifications in part. A multi-step view of autoimmune disease pathogenesis indicates that tolerance checkpoints exist and that the genes and molecular pathways that underpin these mechanisms overlap with those involved in tumour suppression.^10^ Elimination or disruption of these pathways can therefore potentially result in the development of both diseases in tandem or sequentially.

### Immunogenetic and Cytokine Response Perspectives

BD straddles the interphase between autoinflammatory and autoimmune diseases. HLA-B*51 alleles are implicated in the immunogenetics of BD.^11^ Of the more than 89 different subtypes of HLA-B51, HLA-B5101 is the major sub-allele associated with BD in most populations studied to date. In Middle Eastern, Italian, Spanish, Greek, Turkish, and German patients, BD is strongly associated with HLA-B5108. Several other HLA class I and II alleles including HLA-A26, HLA-B15, HLA-B5701, HLA-B2702, HLA-B3901, HLA-B52, HLA-B56, Cw1, Cw14, Cw15, Cw16, HLA-DRB104, and HLA-DRB107 have been shown to be linked to BD in other populations. The overall effect of these genes which influence the adaptive and innate immunity systems, is to alter the T-cell repertoire, inducing polarisation towards the Th1/Th17 profile in BD.^12^

Individual amino acid residues located on HLA-B51 molecules are associated with disease and are located in the antigen binding regions mediating peptide binding and interactions between CD8 lymphocytes, Natural killer (NK) cells and MHC class I (MHC-1) molecules.^12^ Studies have also shown an association between BD and the MHC Class I polypeptide-related sequence A (MICA) allele 009. MICA are genes found on MHC-1 region in the chromosome which code for proteins expressed on cells such as endothelium and fibroblasts. However, the significance of this association is not known; this association could just be linkage disequilibrium between MICA and HLA-B51,^13^ while the recent Genome-Wide Association Study (GWAS) did not find an independent association.^14^ A recent GWAS has demonstrated that expression of risk alleles in BD leads to defects in cytokine gene expression that results in decreased expression of the anti-inflammatory cytokine IL-10, which can down-regulate the expression of pro-inflammatory cytokines such as TNF α, IL-6 and IL-12; inhibition of the costimulatory coupling activities of macrophages on T cell and NK cells; and the expression of disease associated variants of IL-23 gene (this regulates Th17 cell development).^14^ IL-12 and IL-23, which are both crucial in Th17-associated pathology such as BD, share the receptor subunit p40 which can be targeted by the monoclonal antibody (Ustekinumab). This biodrug is effective in disease amelioration.^15^ It could therefore be postulated that this drug could also be a potential effective therapy for BD.

Pro-inflammatory cytokine IL-6 is essential for the growth of human B lymphocytes and myeloma cells, whilst TNFα plays a role in the pathogenesis of plasma cell dyscrasias.^16^ Serum IL-6 levels are significantly elevated in MGUS compared to controls.^17^ Similar observations have been made for TNFα and IL-8. However, the levels of TNFα and IL-8 were not significantly associated with a higher probability of malignant transformation as previously reported.^18^ Furthermore, Zheng et al. found no association between polymorphisms of IL-6, TNFα, and MGUS.^16^ Serum IL-6 and TNFα have been shown independently, to be significantly elevated in patients with BD when compared to health controls, and their levels correlate with BD disease activity.^17^

IL-10 levels are decreased in BD whilst the expression of IL-6 and TNFα are upregulated. Raised levels of IL-6 might promote aberrant plasma cell growth, which may play a role in the pathophysiology of MGUS by promoting the growth and survival of myelomatoid cells. A potential unifying early event (e.g., IgH translocation) may render B cells vulnerable to proliferative stimuli such as IL-6. IL-6 plays an essential role in plasma cell disorders and more importantly progression to Multiple Myeloma (MM).^18^

IL-6 induced stimulation and proliferation of cells requires signal transduction mediated by the STAT (Signal Transducers and activators of transcription) (STAT1 and STAT3) and MAPK (mitogen-activated protein kinase) pathways.^19^ In inflammation, a positive feedback auto-crine loop exists in fibroblasts with upregulated STAT4 leading to sustained IL-6 transcription.^20^ Whether fibro-blasts are involved in BD or MGUS pathogenesis remains to be determined.

Risk alleles rs897200, rs7564070 and rs7572482 in BD are associated with raised expression of STAT4 gene and upregulation of the Th17 pathway. STAT4 gene encodes the transcription activator and signal transducer STAT4, which is activated by proinflammatory cytokines such as IL-12 and plays a role in T-cell maturation.^21^ STAT4 has been identified through GWAS as a disease susceptibility loci shared in several immune diseases such as BD, RA and SS.^12^ STAT5, an anti-apoptotic transcription activator which acts directly on the IL-17 gene to limit IL-17 transcription, is in a balance with STAT3 (pro-inflammatory promoting IL-17 production) and variation in the levels of cytokines activating these factors determine the outcome of the activities of Th17 cells.^15^

The overlap of susceptibility genes therefore, suggests an overlap of pathophysiogenetic mechanisms. SS, SLE, RA and spondyloarthropathies have been associated with MGUS.^4^ However seropositive rheumatic arthritis is strongly associated with MHC class II whilst seronegative diseases and BD show an association with MHC class I.^22^

### Immune System Interaction Considerations

CD8^+^ T cells, T regulatory (Treg) and Th17 cells are implicated in BD pathogenesis. CD8+ and CD56^+^ T cells are increased in the peripheral blood and aqueous humour of BD patients with uveitis.^23^ These cells produce IFN-γ in active disease which upregulates and augments their cytolytic tissue destructive effects.^24^ The cytolytic and effector functions of CD8+ cells correlate with CD56 cellular expression.^25^ Chronic antigen stimulation induces CD56 expression on cytotoxic T-lymphocytes in BD.^25^ This surface marker is also expressed by aberrant CD56+ plasma cells in MGUS which act as clonal cells.^1^ The CD8+ T cell population in patients with MGUS is significantly expanded with a high degree of clonality.^26^ These expansions were attributed to chronic antigen stimulation. Such shared immune pathways might explain the development of MGUS in our BD patient.

The disease alleles in BD are in antigen binding regions which mediate interactions between CD8+ and MHC class I molecules. An abnormal response to antigen stimulation could promote a Th1 dominant microenvironment with enhanced CD8+ T cell cytotoxic effects due to excessive IFN-γ production. We propose that chronic antigen stimulation may sustain this response in BD and this coupled with dysregulated cytokine microenvironment -induced cytogenetic abnormalities, could initiate and/or maintain proliferation of aberrant clonal CD56+ and CD8+ T cells which promote MGUS.

BD patients with active and untreated disease have high blood circulating levels of Th17 cells and low levels of Treg cells mediated by IL-21 which correlates with disease activity.^27^ IL-21 is produced by CD4^+^ T-cells.^12^ Activated Th17 cells produce IL-17 under the influence of IL-6 produced after antigenic (extracellular pathogens) activation of the innate immune system, which upregulates adhesion molecule expression on endothelial cells.^28^ Therefore, enhanced Th17 cellular activity plays a role in the vascular inflammation and thrombosis in BD. There is a complex functional antagonism between Treg and Th17 cells in chronic inflammation. However, studies on the role of Treg cells in BD have produced conflicting results.^27,29^ Tregs have an essential role in peripheral tolerance and the preservation of self-tolerance.^30^ The clinical manifestation of BD support decreased numbers and function of Tregs in active disease.

A balance between Treg and Th17 is essential for maintaining anti-tumour immunity, and IL-6 plays a pivotal role in regulating this balance. The role of Treg in neo-plastic disease is also plagued by contradictory results as seen in BD. A similar reduction of Treg has been reported in peripheral blood from patients with MGUS.^30^ However, other studies have shown no significant difference in comparison to normal controls.^31^ An increase in Th17 cells has been demonstrated in the bone marrow of patients with multiple myeloma but not in those with MGUS.^32^

Although there is limited research into the Th1/Th2 ratio in myeloma patients, a study to determine the clinical significance of this ratio found an insignificant increase in the ratio in MGUS patients.^33^

### Environmental Perspectives

Immunogenetics provide insight into the means of predisposition but does not completely account for incidence of BD. There is interestingly, a significant carriage rate of HLA-B51 in healthy individuals.^34^ Chronic antigen stimulation or abnormal response to antigen stimulation has been implicated in the pathogenesis of both BD and MGUS.^12,35^ BD commonly begins in oral mucosa, and oral lesions increase after dental work, with reports of antibacterial therapy decreasing symptoms; supporting the potential role of microbial flora possibly mediated by non-specific T-cell hyperactivity against the ubiquitous antigens.^8^ Expression of risk alleles of chemokine receptor and H antigen encoding genes alter the body’s response to microbial pathogens increasing BD risk.^12^

High levels of Heat shock proteins (HSP60) have been reported in BD and RA, where they can act as a danger signal of abnormal antigen presentation.^34^ HSPs are synthesised in response to cell exposure to non-specific stimuli such as infection or trauma. HSP60 acts as a ligand for Toll-Like-Receptors (TLR), stimulating inflammatory cytokines (IL12, IL6, TNFα) release which can boost adaptive Th1 immune responses. Subsequent activation of the innate adaptive immunity fits in with the clinical spectrum of BD.^36^ Therefore, the pathogenesis of BD can putatively be viewed as an antigen-driven immune response superimposed on the primed state present in predisposed individual, induced by heat shock proteins or other non-specific antigens.^37^

The racial predisposition in MGUS could indicate the influence of environmental factors rather than genetics.^18^ The pathogenesis of MGUS can on the other hand be hypothesised to be an abnormal response to antigenic stimulation, mediated possibly by aberrant expression of toll-like receptors and overexpression of IL-6 bioactivity.^35^

### Epigenetics

The role of epigenetics in the pathogenesis of BD and its association with MGUS could explain in part the contribution of the environment to the pathogenetic mechanisms. MicroRNAs which are short noncoding RNAs that negatively regulate gene expression by acting post-transcriptionally, have been implicated in BD.^38^ They are crucial in modulating cellular processes such as proliferation and apoptosis. MicroRNA-155 is significantly decreased in dendritic cells from BD patients whilst IL-6 and IL-17 production are increased.^39^ DNA methylation - an epigenetic mechanism through which methyl groups are added to DNA molecule causing genes to switch off or on - were identified by a recent genome-wide study as playing a role in the epigenetic remodelling of cytoskeleton-related genes involved in the pathogenesis of BD.^40^ Dysregulation of these genes could underpin increased leukocyte migration observed in BD. In comparison to health controls, 283 and 125 differentially methylated sites were identified in monocytes and CD4+ T-cells from BD patients. Interestingly, treatment reversed these methylation differences with complete restoration of a normal profile in many cases.^40^

GWAS has shown changes in DNA methylation in MGUS also, with aberrant demethylation occurring in Cyto-sine-phosphodiester-Guanine (CpG) islands and differential methylation occurring in genes involved in cell proliferation and cell cycle.^41^ A microRNA microarray study has reported upregulation of microRNA with oncogenic functions, such as miR-21 in MGUS, which could promote plasma cell transformation by blocking apoptosis. Upregulation of miR-17 downregulates a gene (SOCS-1) which play a critical role in inhibiting IL-6 growth signalling.^42^ Epigenetic therapy targeting the distinctive signatures in MGUS, myeloma and BD inflammatory cells could therefore have therapeutic potential.

### Therapeutic Implications

BD and MGUS have shared and specific pathogenetic mechanisms which are important targets for current and emerging therapies. Potential therapeutic targets such as biologics are avenues for further exploration and for development of personalised therapy. Cytokine profiling (e.g. elevated levels of 1L-1β, 1L-12, 1L-17) can aid in the identification of which biologicals are potential treatments based on the cytokine profile of a disease. Microarray gene profiling will enable assessment of gene expression patterns that may help identify which patients can respond to certain targeted therapy such as epigenetic therapy. There is also a role in monitoring to detect changes in cytokine or epigenetic profiles as a measure of disease remission or as a signal to change to a better suited therapy.

## CONCLUSION

Chronic antigen stimulation in genetically predisposed individuals leads to a sustained inflammatory response in BD leading to perturbation of anti- and pro-inflammatory cytokine and T-cell balances. The resultant inflammatory microenvironment may promote the development of aberrant clonal plasma cells leading to MGUS. Epigenetic contributions can perpetuate proliferative and anti-apoptotic mechanisms, promoting disease development. A more developed understanding of the disease pathogenetic mechanisms linking BD and MGUS may provide further insights into the aetiopathophysiology and create options for new and targeted therapeutics in BD and possibly MGUS.
